# Effect of the combination of acupuncture based on Yuan-Luo Da-Jie-Jing and repetitive transcranial magnetic stimulation on type 1 complex regional pain after stroke: a randomized controlled trial protocol

**DOI:** 10.3389/fneur.2025.1702965

**Published:** 2025-12-10

**Authors:** Huan Liu, Xin-yuan Zhang, Kun Hong, Juan Gao

**Affiliations:** 1 Rehabilitation Department, Nanbu County People’s Hospital, Nanchong, China; 2 Police Academy, Chinese Armed Police Force, Chengdu, China; 3 Department of Acupuncture and Moxibustion, No. 3 Affiliated Hospital of Chengdu University of Traditional Chinese Medicine (West District), Chengdu, China; 4 Medical Service Training Center, General Hospital of the Western Theater of the Chinese People’s Liberation Army, Chengdu, China

**Keywords:** acupuncture, rTMS, stroke, complex regional pain, protocol

## Abstract

**Background:**

Stroke is a major cause of death and disability worldwide. Approximately 12–25% of stroke survivors develop complex regional pain syndrome type I (CRPS-I) within 3–12 months. CRPS-I is characterized by severe limb pain, abnormal pain, and vasomotor instability, which hinders rehabilitation. Insufficient efficacy and adverse reactions limit the use of current pharmacological treatments, such as non-steroidal anti-inflammatory drugs and gabapentinoids. Post-stroke CRPS-I is characterized by neuroinflammation, maladaptive neural plasticity, and central/peripheral sensitization. Repetitive transcranial magnetic stimulation (rTMS) regulates pain through cortical reorganization and inhibition of neuroinflammation, while acupuncture, particularly the Yuan-Luo Dajiejing (YLDJJ) technique, reduces central sensitization by activating endogenous opioids. However, no randomized controlled trial has compared the efficacy of YLDJJ acupuncture combined with rTMS in the treatment of CRPS-I after stroke.

**Methods:**

This randomized controlled trial will randomly assign eligible participants to three groups in a 1:1:1 ratio: the experimental group (YLDJJ acupuncture + rTMS), the control group 1 (conventional acupuncture + rTMS), and the control group 2 (sham acupuncture + rTMS). The participants will be aged 18–80 years old, with CRPS-I developing after stroke and meeting the specific inclusion criteria. The intervention measures will be administered once daily for 20 days. Pain intensity, assessed using the visual analogue scale (VAS), will be measured at baseline, on days 10 and 20, and at 1 and 3 months after treatment completion. Secondary outcome measures include motor function, muscle tone, shoulder range of motion, activities of daily living, thermal asymmetry, and neuroplasticity-related indicators. The sample size will be 126 participants, accounting for a 20% dropout rate.

**Conclusion:**

This trial will evaluate the efficacy and safety of Yuan-Luo Da-Jie-Jing acupuncture combined with rTMS. The results will be compared to two control groups: one receiving conventional acupuncture with rTMS, and the other receiving sham acupuncture with rTMS. The results are expected to provide evidence for formulating clinical treatment strategies for CRPS-I after stroke.

**Clinical trial registration:**

(ITMCTR, http://itmctr.ccebtcm.org.cn/), No. ITMCTR2025001677.

## Introduction

Stroke, including ischemic and hemorrhagic subtypes, is the second leading cause of mortality and a major contributor to long-term disability globally. It imposes a substantial socioeconomic burden due to acute neurological deficits and chronic sequelae (1). While the incidence rates of stroke increase steadily with age and roughly double every 10 years after 55 years of age, both ischemic (2) and hemorrhagic strokes are becoming increasingly more prevalent among younger people (3). One major complication of stroke is complex regional pain syndrome type I (CRPS-I), affecting 12–25% of stroke survivors within 3–12 months (4). Characterized by severe limb pain, allodynia, and vasomotor instability (5), CRPS-I profoundly undermines recovery and quality of life.

The pathogenesis of CRPS-I involves neuroinflammation and maladaptive plasticity (6). Stroke-induced injury triggers peripheral sensitization via cytokines and sympathetic-pain coupling (7–9), leading to central sensitization (7, 10) and cortical reorganization (11). Autonomic dysfunction, including vasoconstriction and catecholamine hypersensitivity (12, 13), and sustained neuroinflammation perpetuate pain (9, 14, 15).

Current pharmacological agents, such as nonsteroidal anti-inflammatory drugs (NSAIDs) and corticosteroids, provide inadequate efficacy (30–40% failure) and several side effects (16, 17). It brings huge challenges to patients’ recovery and treatment.

Repetitive transcranial magnetic stimulation (rTMS), specifically high-frequency rTMS targeting the contralateral M1 (level C recommendation) (18), shows promise for the treatment of stroke. Its analgesic mechanisms include modulation of the sympathetic system, restoration of cortical representation, suppression of neuroinflammation (19, 20), and attenuation of central sensitization (21), promoting adaptive plasticity (22, 23).

Acupuncture alleviates central sensitization via endogenous opioid release and modulation of neuroinflammatory cascades (24). Specific techniques, such as YLDJJ, may be effective for post-stroke neuropathic pain (25, 26). Diverging from conventional local acupoint selection, YLDJJ, derived from the classical doctrine of ‘12 meridians connecting sequentially as an endless cycle’ (Ling Shu·Meridians), emphasizes systemic neuroregulation through sequential needling of Yuan-Source (primary qi-influx loci) and Luo-Connecting (inter-meridian anastomosis loci) points on all 12 meridians. It aims to restore global qi-blood dynamics (a concept in traditional Chinese medicine). Contemporary evidence suggests three technical distinctions: (1) Holistic modulation: YLDJJ is proposed to augment functional connectivity between perilesional sensorimotor cortex and contralateral cerebellum (27), unlike conventional peri-shoulder acupuncture, which preferentially activates ipsilateral somatosensory cortices; (2) anti-neuroinflammatory specificity: Animal studies have shown that YLDJJ may downregulate Reg3b expression in dorsal root ganglia (2.1-fold greater than conventional acupuncture), disrupting TNF-*α*-mediated neuro-immune crosstalk (28); (3) sustained analgesia: Previous RCTs using YLDJJ have reported 19.7% superior pain relief retention at 3 months compared to local acupoint protocols for CRPS-I (25). These mechanistic properties may enable YLDJJ to disrupt the “peripheral sensitization, central maladaptation, and autonomic dysfunction” vicious cycle in CRPS-I. It also enhances pain thresholds, neural reorganization (27, 29), corticospinal tract remodeling, and cerebral blood flow (30). Nevertheless, acupuncture phobia and protracted treatment regimens undermine adherence (31).

Based on the aforementioned hypothesis regarding the regulatory effect of rTMS on central sensitization and the assumption that YLDJJ acupuncture can systematically modulate the pathological progression of CRPS-I, this approach not only addresses the insufficient efficacy of rTMS alone but also overcomes the low compliance of patients with traditional acupuncture.

## Objectives

Regarding the treatment of post-stroke CRPS-I, the relative efficacy of three interventions, including YLDJJ acupuncture combined with rTMS, conventional acupuncture combined with rTMS, and sham acupuncture plus rTMS, remains inadequately validated. Furthermore, no RCT has evaluated the efficacy and safety of these interventions in patients suffering from post-stroke CRPS-I. Therefore, we designed this RCT protocol to assess the effects of the three interventions on post-stroke CRPS-I and its associated symptoms, aiming to provide evidence-based guidance for the clinical management of post-stroke CRPS-I.

## Methods

### Trial design

**Figure 1:**
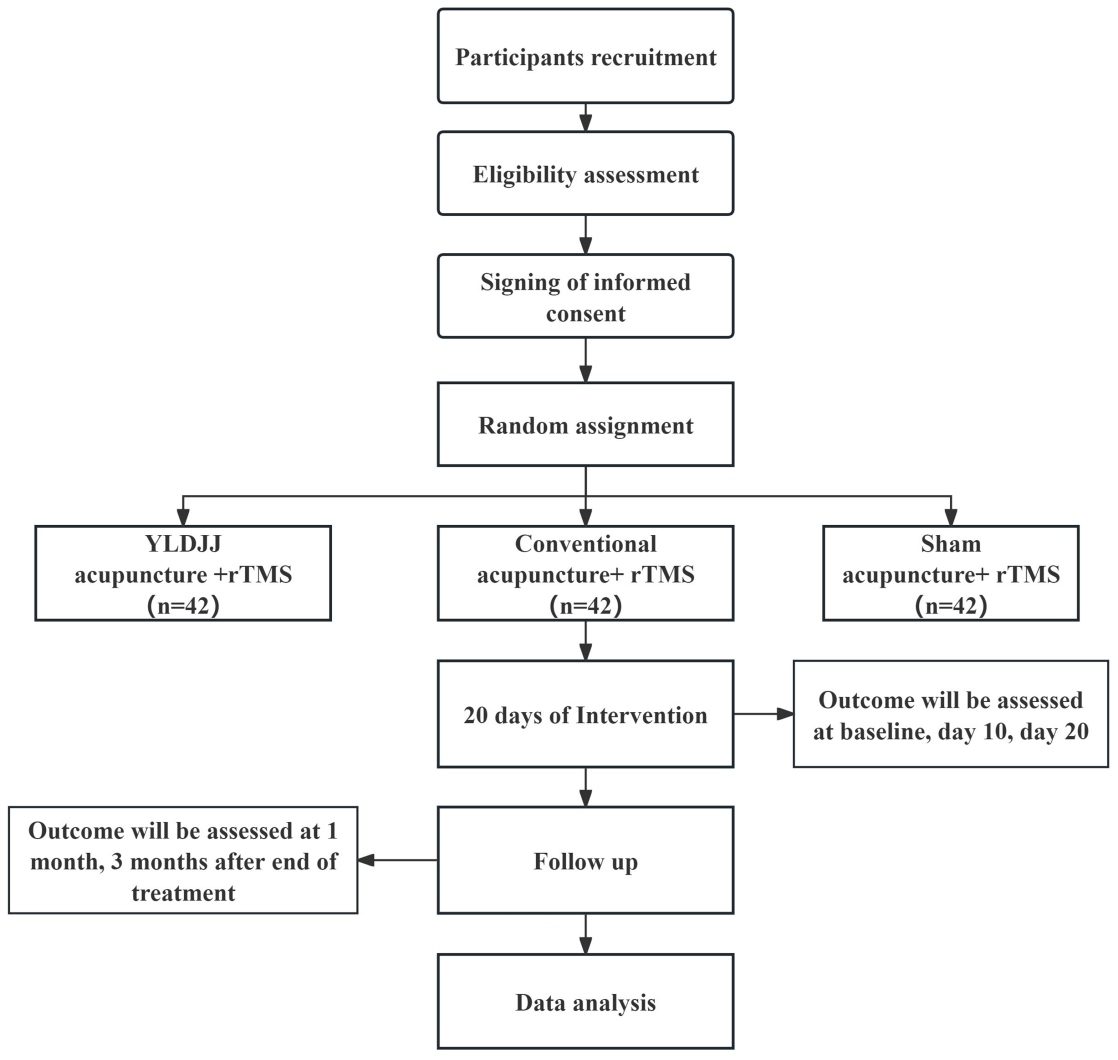
Flow chart for clinical trial procedure.

This trial protocol adheres to the complete list of the SPIRIT 2025 Statement (32). A trial flowchart was created to clearly present the entire implementation process of this RCT. Details can be found in Figure 1. The diagram covers participant recruitment and screening, randomization, intervention implementation, outcome assessment, and data analysis. It also visualizes the intervention pathways and key time points of the three groups.

### Study participants

This RCT will be conducted at Nanbu County People’s Hospital, following the Guidelines for the Diagnosis and Initial Management of Stroke and Transient Ischaemic Attack in Individuals Aged 16 Years and Over (33), and the Budapest Criteria for CRPS-I diagnosis (4). Specialist physicians will assess the eligibility of potential participants. Those who voluntarily participate will sign an informed consent form before enrollment.

### Eligibility criteria

#### Inclusion criteria

(1) Confirmed diagnosis of CRPS-I based on the Budapest clinical diagnostic criteria.
(2) CRPS-I secondary to ischemic or hemorrhagic stroke confirmed based on CT/MRI within 3–12 months before enrollment.
(3) Mean pain intensity ≥4 on the visual analogue scale (VAS; 0–10 cm) during 7 days before the intervention.
(4) Unilateral upper-limb pain localized to the hemiparetic extremity.
(5) Neurologically stable status, defined as a National Institutes of Health stroke scale (NIHSS) score ≤15.
(6) Adequate tolerability for both acupuncture and repetitive rTMS interventions.
(7) Aged between 18 and 80 years at screening.
(8) Documented hand dominance before stroke.
(9) Provision of written informed consent voluntarily obtained before participating in the trial.

#### Exclusion criteria

(1) Diagnosis of type 2 CRPS or a documented history of CRPS before stroke.
(2) Presence of current limb fracture, local infection, or peripheral neuropathy in the affected extremity.
(3) Presence of severe aphasia, unilateral neglect, or cognitive impairment.
(4) History of recurrent stroke, brainstem or cerebellar lesions, or bilateral involvement.
(5) Presence of implanted metal or electronic medical devices, such as cardiac pacemakers, a history of epilepsy, or intracranial hypertension.
(6) Bleeding problems, fear of needles, or skin problems where acupuncture will be done.
(7) Presence of comorbid central pain after stroke, phantom limb pain, or other chronic pain syndromes.
(8) Presence of severe psychiatric disorders.
(9) Opioid use or undergoing interventional treatment for pain within 4 weeks before enrollment.
(10) Pregnancy, lactation, or concurrent participation in other clinical trials.

### Criteria for discontinuation of the intervention

Participant involvement in the trial may end early due to the following reasons:

Serious adverse event (SAE): Discontinue immediately if the investigator links an SAE to acupuncture or rTMS.

Withdrawal of consent: Participants may voluntarily withdraw consent at any time without a reason.

Protocol violation: Major deviations that compromise safety or data integrity, as judged by the principal investigator (e.g., starting prohibited treatments or finding ineligibility after randomization).

Loss to follow-up: Participant cannot be reached for three sessions or assessments.

Clinical deterioration: An independent clinician confirms a decline in health status, making participation unsafe or impractical.

Investigator’s decision: The investigator may stop intervention for any reason, protecting the participant’s best interest.

For all participants discontinuing the treatment, the reason and the date of withdrawal will be recorded in the case report form. We attempt to assess the final outcome at discontinuation.

### Criteria for exclusion from analysis

All randomized participants will be included in the primary intention-to-treat (ITT) analysis. The criteria for the per-protocol (PP) population and data handling are mentioned in the following sections:

### Exclusion from PP analysis

Participants will be excluded from the PP analysis if they undergo fewer than 10 intervention sessions (50% of the planned treatment) without a medically justified reason. They will be excluded because insufficient exposure prevents the assessment of treatment efficacy.

Major protocol violations that critically affect efficacy evaluation, such as incorrect randomization, receiving the wrong intervention group assignment, or violation of the key inclusion/exclusion criteria, will also lead to exclusion from PP analysis.

Additionally, missing the primary outcome (VAS score) at the post-treatment (day 20) time point results in exclusion from the PP analysis.

### Handling of missing data

For the primary ITT analysis, missing data will be handled using multiple imputation by chained equations (MICE). The details are described in the statistical plan.

Sensitivity analyses will be used to compare the results of the imputed ITT population with those of the complete-case PP population to assess the robustness of the results.

### Interventions

#### Quality control for acupuncture

All acupuncturists have at least 10 years of clinical experience and possess 40 h of standardized training on both YLDJJ and conventional acupuncture protocols, including video-based assessment for point selection and needling technique. For quality control, a third-party expert will randomly select 10% of sessions to verify consistency. Additionally, separate personnel will be assigned to provide daily treatment reminders to the participants.

#### Experimental group: Yuan-Luo Da-Jie-Jing (YLDJJ) acupuncture + rTMS

**Figure 2:**
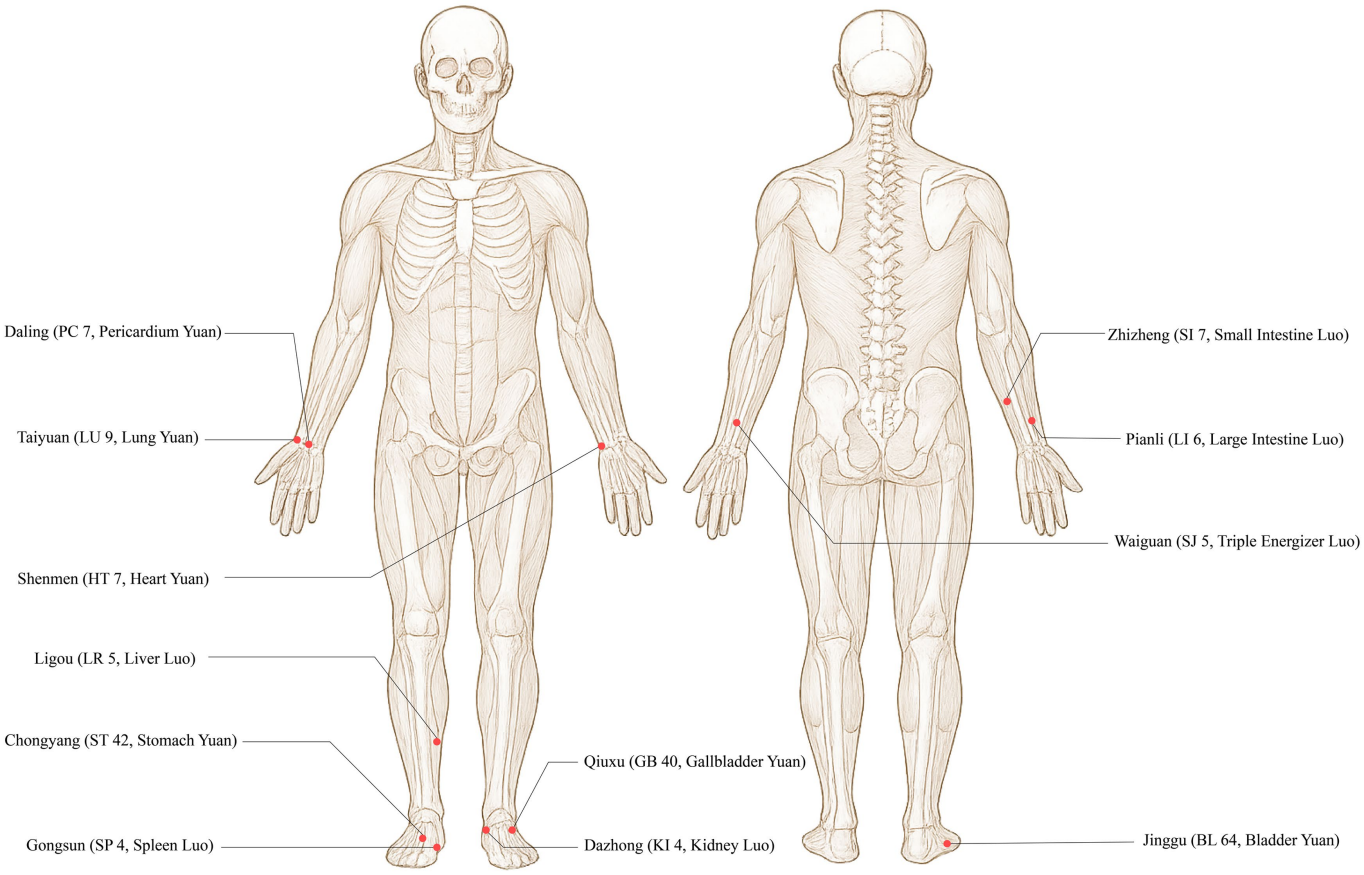
Acupoint illustration for YLDJJ acupuncture.

The acupuncture procedure will be conducted based on the following sequence:

Acupoint selection and sequence: Yuan-Source points and Luo-Connecting points of the 12 regular meridians will be needled sequentially, with alternation between affected and unaffected sides. The specific order is as follows: Taiyuan (LU 9, Lung Yuan-Source) → Pianli (LI 6, Large Intestine Luo-Connecting) → Chongyang (ST 42, Stomach Yuan-Source) → Gongsun (SP 4, Spleen Luo-Connecting) → Shenmen (HT 7, Heart Yuan-Source) → Zhizheng (SI 7, Small Intestine Luo-Connecting) → Jinggu (BL 64, Bladder Yuan-Source) → Dazhong (KI 4, Kidney Luo-Connecting) → Daling (PC 7, Pericardium Yuan-Source) → Waiguan (SJ 5, Triple Energizer Luo-Connecting) → Qiuxu (GB 40, Gallbladder Yuan-Source) → Ligou (LR 5, Liver Luo-Connecting). The location and attributes are detailed in Table 1 and Figure 2.

**Table 1:** Acupoint localization and attributes for YLDJJ acupuncture.

Acupoint name	Code	Meridian	Specific point type	Localization (WHO standard)
Taiyuan	LU 9	Lung	Yuan-source	On the wrist, at the radial end of the transverse crease, in the depression lateral to the radial artery.
Pianli	LI 6	Large intestine	Luo-connecting	On the forearm, 3 cun proximal to LI5 (Yangxi), on the line connecting LI5 and LI11, between the radius and ulna.
Chongyang	ST42	Stomach	Yuan-source	On the dorsum of the foot, between the 2nd and 3rd metatarsal bones, in the depression distal to the base of the 2nd metatarsal bone, over the dorsalis pedis artery.
Gongsun	SP 4	Spleen	Luo-connecting	On the medial foot, distal and inferior to the base of the 1st metatarsal bone, in the depression at the border of the red and white skin.
Shenmen	HT 7	Heart	Yuan-source	On the wrist, at the ulnar end of the transverse wrist crease, in the depression radial to the tendon of the flexor carpi ulnaris.
Zhizheng	SI 7	Small intestine	Luo-connecting	On the forearm, 5 cun proximal to the dorsal wrist crease, on the line connecting SI5 (Wangu) and SI8 (Xiaohai), between the ulna and the tendon of the flexor carpi ulnaris.
Jinggu	BL 64	Bladder	Yuan-source	On the lateral foot, distal to the tuberosity of the 5th metatarsal bone, at the junction of the red and white skin.
Dazhong	KI 4	Kidney	Luo-connecting	On the medial foot, posterior and inferior to the medial malleolus, in the depression anterior to the Achilles tendon.
Daling	PC 7	Pericardium	Yuan-source	On the wrist, at the midpoint of the transverse wrist crease, between the tendons of the palmaris longus and flexor carpi radialis.
Waiguan	SJ 5	Triple energizer	Luo-connecting	On the forearm, 2 cun proximal to the dorsal wrist crease, between the radius and ulna.
Qiuxu	GB40	Gallbladder	Yuan-source	On the dorsum of the foot, anterior and inferior to the lateral malleolus, in the depression lateral to the tendon of the extensor digitorum longus.
Ligou	LR 5	Liver	Luo-connecting	On the leg, 5 cun above the tip of the medial malleolus, on the medial border of the tibia.

cun: Relative measurement based on the patient’s anatomy (e.g., the width of the thumb at the interphalangeal joint = 1 cun). Radial/Ulnar: Thumb side (radial)/little finger side (ulnar) of the arm. Medial/Lateral: Toward the midline (medial)/away from the midline (lateral) of the body. Proximal/Distal: Closer to the trunk (proximal)/farther from the trunk (distal).

#### Acupuncture technique

Sterilized disposable stainless steel needles (0.30 mm × 40 mm, Hwato) will be employed. The insertion depth will range from 10 to 25 mm based on anatomical location. A standardized reinforcing-reducing manipulation will be applied, involving uniform lifting, thrusting, and rotating maneuvers at 60–90 cycles per minute for 20–30 s per acupoint to achieve deqi.

Deqi definition: Deqi is defined as a combination of subjective sensations reported by the patient (e.g., soreness, numbness, heaviness, and distension) and the objective sensation of needle grasp felt by the practitioner (34, 35). Needle retention: Needles will remain *in situ* for 30 min.

Treatment schedule: Sessions will be conducted once daily, 5 days per week, for a total of 20 sessions over 4 weeks. Each session will last approximately 40 min.

**Figure 3:**
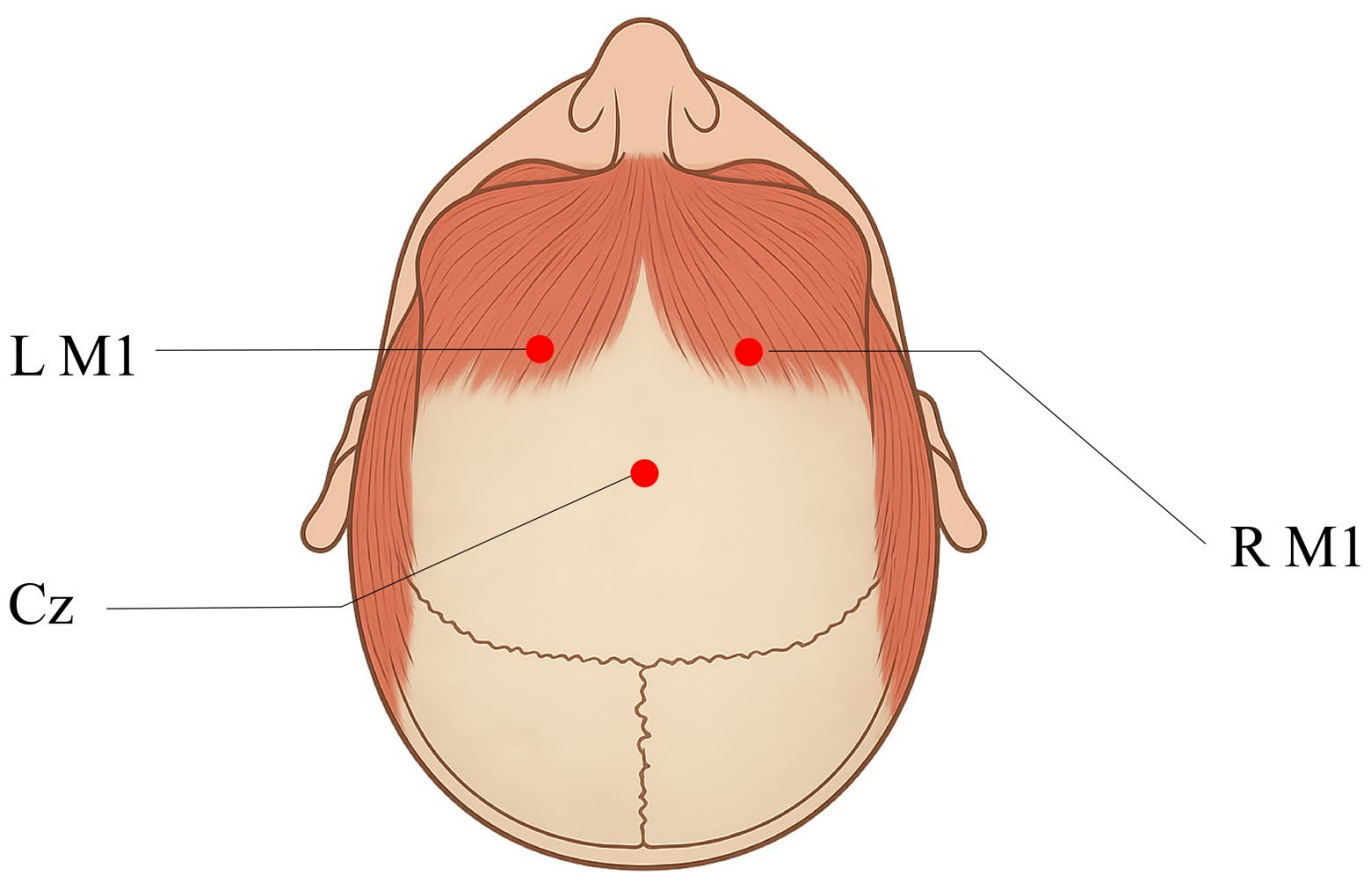
Schematic diagram of rTMS targeting the primary motor cortex (M1 area). L M1 represents the left primary motor cortex, R M1 represents the right primary motor cortex. The M1 area is the core region of the brain responsible for regulating body movements. CZ is the standard electrode position in scalp electroencephalography (central zero point), located on the midline of the scalp, approximately corresponding to the central area of the top of the head, and can be used as one of the anatomical reference landmarks for locating the M1 area.

For rTMS, stimulation will be delivered to the contralateral M1 hand area using the Shenzhen Yingzhi M-50 apparatus. The M1 hand area will be identified using the international 10–20 EEG system for scalp electrode placement, with the target site at C3 (for right hemiparesis) or C4 (for left hemiparesis). The resting motor threshold (RMT) will be determined before the first treatment session to individualize stimulation intensity. Key parameters, including frequency, intensity, and pulse configuration, are summarized in Table 2. The schematic diagram of M1 is shown in Figure 3.

**Table 2:** Parameters for repetitive rTMS protocol.

Parameter	Specification
Device	Shenzhen Yingzhi liquid-cooled circulating M-50 Ultimale rTMS therapeutic apparatus
Stimulation site	Contralateral primary motor cortex hand area corresponding to the painful limb(M1)
Frequency	10 Hz
Intensity	80–120% of resting motor threshold
Trains per session	20
Pulses per train	1 pulses per train
Train duration	10 s
Inter-train interval	10 s
Total pulses/session	2,000
Session duration	19 min 50 s
Treatment schedule	Once daily for 20 consecutive days

#### Control group 1: conventional acupuncture (CA) + rTMS

**Figure 4:**
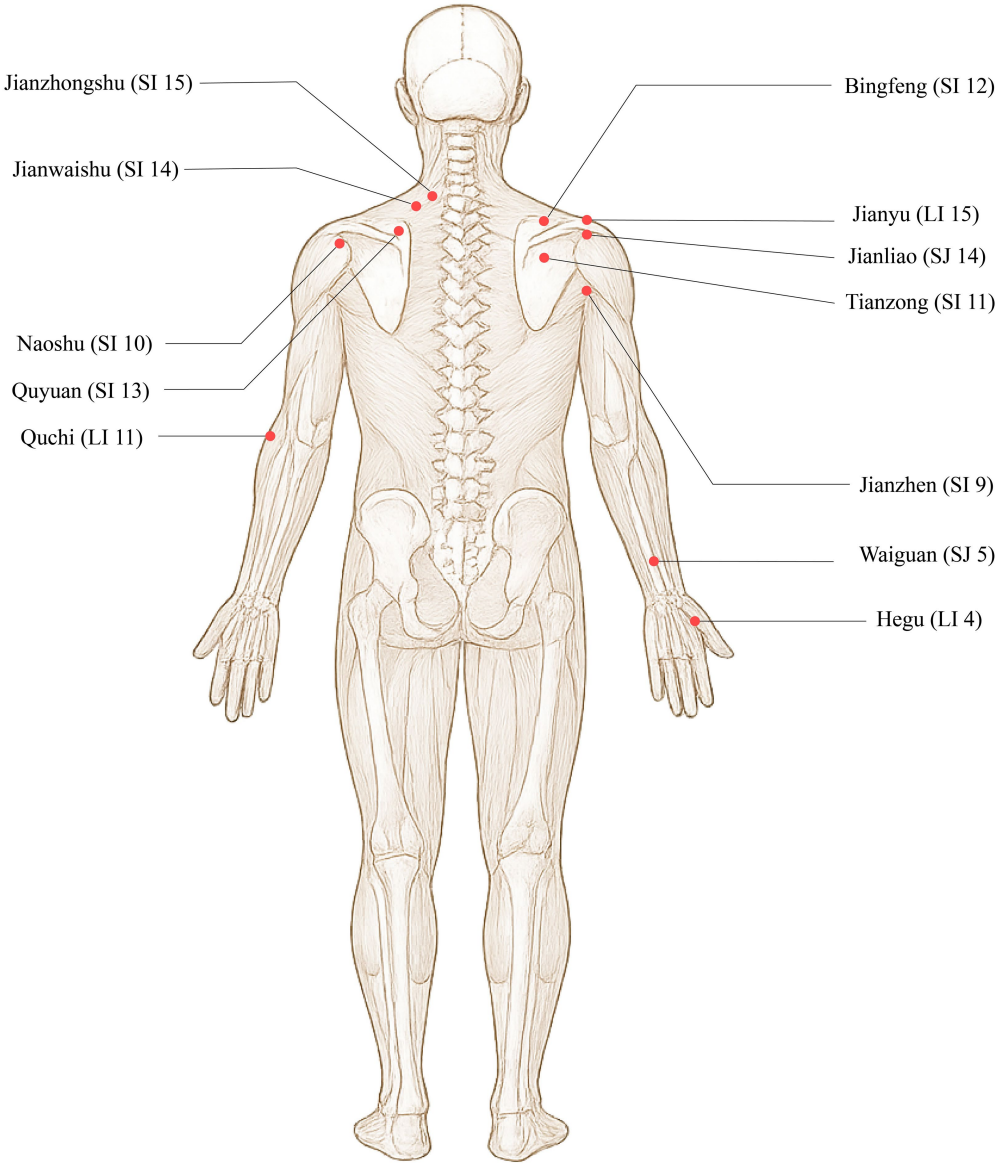
Acupoint illustration for conventional acupuncture.

Conventional acupuncture acupoints are as follows:

Jianyu (LI 15), Jianliao (SJ 14), Jianzhen (SI 9), Naoshu (SI 10), Bingfeng (SI 12), Tianzong (SI 11), Quyuan (SI 13), Jianwaishu (SI 14), Jianzhongshu (SI 15), Quchi (LI 11), Waiguan (SJ 5), and Hegu (LI 4). The locations are detailed in Table 3 and Figure 4.

**Table 3:** Acupoint localization for conventional acupuncture.

Acupoint name	Code	Meridian	Anatomical localization (WHO standard)
Jianyu	LI 15	Large intestine	On the shoulder, anterior and inferior to the acromion, in the depression formed when the arm is abducted to the horizontal position.
Jianliao	SJ 14	Triple energizer	On the shoulder, posterior to the acromion, in the depression about 1 cun posterior to LI15, midway between LI15 and SI9.
Jianzhen	SI 9	Small intestine	On the posterior shoulder, 1 cun superior to the posterior axillary fold when the arm is adducted, in the depression inferior to the scapular spine.
Naoshu	SI 10	Small intestine	On the scapula, in the depression superior to the posterior axillary fold, directly above SI9 at the level of the axillary crease.
Tianzong	SI 11	Small intestine	In the center of the supraspinous fossa of the scapula, directly superior to SI11, midway between SI10 and SI13.
Bingfeng	SI 12	Small intestine	In the center of the infraspinous fossa of the scapula, at the midpoint between the inferior border of the scapular spine and the inferior angle of the scapula.
Quyuan	SI 13	Small intestine	Medial to the scapular spine, at the midpoint between SI10 (Naoshu) and the spinous process of the 2nd thoracic vertebra (T2).
Jianwaishu	SI 14	Small intestine	On the back, 3 cun lateral to the lower border of the spinous process of the 1st thoracic vertebra (T1).
Jianzhongshu	SI 15	Small intestine	On the back, 2 cun lateral to the lower border of the spinous process of the 7th cervical vertebra (C7).
Quchi	LI 11	Large intestine	At the lateral end of the cubital crease, midway between LU5 (Chize) and the lateral epicondyle of the humerus, with the elbow flexed.
Waiguan	SJ 5	Triple energizer	On the forearm, 2 cun proximal to the dorsal wrist crease, between the radius and ulna.
Hegu	LI 4	Large intestine	On the dorsum of the hand, between the 1st and 2nd metacarpal bones, at the midpoint of the 2nd metacarpal bone on the radial side.

Acromion: Bony prominence at the lateral end of the scapular spine; Supraspinous: Depression above the scapular spine; Infraspinous: Depression below the scapular spine; cun: Relative measurement based on the patient’s anatomy (e.g., the width of the thumb at the interphalangeal joint = 1 cun); C7: Most prominent cervical spinous process when neck is flexed; T1/T2: First/s thoracic vertebrae below C7.

#### Acupuncture technique

Identical needles (0.30 mm × 40 mm) will be used for acupuncture. The insertion depth, manipulation to achieve deqi, deqi criterion, needle retention time (30 min), and treatment schedule (once daily, 20 sessions) will be standardized to match the procedures in the experimental group, leaving only the acupoint prescription as the major variable.

rTMS procedure:

The rTMS protocol (parameters, site, and schedule) is identical to that described for the experimental group.

#### Control group 2: sham acupuncture + rTMS

Acupuncture technique:

Acupoint selection: The same set of 12 acupoints used in the experimental group will be used for the control group.

Sham needling technique: The non-penetrating Streitberger-type placebo needle (Asiamed) will be applied at each acupoint (36, 37). This device has a blunt tip that retracts into the handle upon contact with the skin, allowing insertion without piercing the skin. The needle will be fixed in place with an adhesive ring to mimic the appearance of a real needle.

Simulated deqi: To maintain blinding, the acupuncturist will gently manipulate the sham needle handle periodically without applying penetrating pressure and will ask open-ended questions about sensation, not mentioning specific deqi sensations.

Needle retention: The sham needles will remain in place for 30 min, matching the retention time in the active acupuncture groups.

Treatment schedule: The session frequency and duration (once daily, 20 sessions) will be identical to the other groups.

rTMS Procedure:

The rTMS protocol (parameters, site, and schedule) will be identical to that described for the experimental group.

### Relevant concomitant care permitted or prohibited during the trial

#### Permitted concomitant care

Patients will be allowed to receive their regular medications for secondary stroke prevention, including antihypertensive drugs, lipid-lowering drugs, hypoglycemic drugs, antiplatelet drugs, and anticoagulants. The dosages of these drugs will remain stable for at least 4 weeks before randomization and shall remain unchanged throughout the entire trial. Celecoxib will be allowed for emergency pain relief, but the dosage, time, and reasons for its use will be recorded in the patient’s diary in a real-time manner. Routine physical therapy of the hemiplegic limb, including range-of-motion training and stretching exercises, will be allowed as long as it begins at least 1 month before enrollment; however, the frequency and intensity of the treatment will not increase in the trial.

#### Prohibited interventions

All pain management interventions beyond the allowed emergency pain relief interventions will be prohibited. This prohibition will cover opioids, neuromodulators (such as gabapentin and pregabalin), corticosteroids, and interventional pain management procedures (such as nerve blocks and spinal cord stimulation). In addition, new pain medications or adjustments to the dosage of existing pain medications will be prohibited. Furthermore, any experimental interventions, such as other forms of neuromodulation, transcranial direct current stimulation, acupuncture, transcutaneous electrical nerve stimulation, and experimental medications or devices, will be prohibited. Invasive interventions, including surgery and joint injections, will also be prohibited. Moreover, intensive rehabilitation interventions for the affected limb, such as constraint-induced movement therapy and robot training, will not be allowed.

#### Outcomes

The efficacy of the intervention will be evaluated by two independent research assistants blinded to group assignment, using pre-specified primary and secondary outcomes. Assessments will be conducted at baseline (before the first treatment), on days 10 and 20, and 1 and 3 months after the end of treatment (Table 4).

**Table 4:** Schedule of outcome assessments across study time points.

Item	Timepoint				
	Baseline	Treatment period		Follow-up period	
		10 day	20 day	1 month	3 month
Enrolment					
Screening	√				
Signed informed consent	√				
Randomization	√				
Intervention					
Yuan-Luo Da-Jie-Jing acupuncture + rTMS		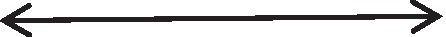			
Conventional acupuncture + rTMS		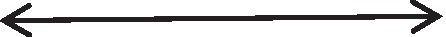			
Sham acupuncture + rTMS		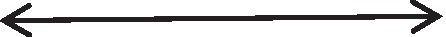			
Assessment					
Primary outcome					
VAS	√	√	√	√	√
Secondary outcome					
Brunnstrom	√	√	√	√	√
Modified ashworth	√	√	√	√	√
AROM	√	√	√	√	√
Simplified Fugl-Meyer	√	√	√	√	√
Improved Barthel index	√	√	√	√	√
Infrared thermal imaging	√	√	√	√	√
RS-fMRI	√	√	√	√	√
CRPS-specific severity	√	√	√	√	√
Other measures					
Blinding assessment		√	√	√	√
Additional treatment		√	√	√	√
Rescue medication use		√	√	√	√
Adverse event		√	√	√	√

VAS, Visual Analogue Scale; rTMS, Repetitive transcranial magnetic stimulation; AROM, active range of motion; RS-fMRI, Resting-state functional magnetic resonance imaging.

#### Baseline

The general condition of eligible participants, including demographic and clinical characteristics, such as age, weight, and disease treatment history, will be recorded in the CRF. The research assistants will conduct the primary and secondary outcomes assessment before the first session of treatment.

#### Primary outcome

Pain intensity will be regarded as the primary outcome. It will be assessed using the visual analogue scale (VAS). The VAS will range from 0 (no pain) to 10 (worst imaginable pain). A reduction of ≥1.5 points will be considered a clinically meaningful improvement (38). The analysis will focus on the mean change from baseline to each time point.

### Secondary outcomes

#### Motor function recovery

Motor function recovery will be assessed using two measures. The Brunnstrom stages of recovery will be used to evaluate upper limb and hand function, ranging from Stage I (flaccidity) to Stage VI (near-normal coordination). The simplified Fugl-Meyer assessment (FMA) will be used to assess the motor function of the upper extremity, with scores ranging from 0 to 66 (higher scores indicating better function).

#### Muscle tone

Muscle tone will be assessed using the modified Ashworth scale (MAS), which measures resistance to passive movement. Scores will range from 0 (no increase in tone) to 4 (rigidity).

#### Shoulder mobility

Shoulder mobility will be evaluated using active range of motion (AROM) for flexion, extension, and abduction. A goniometer will be used, with a range of 0° to 180°. Higher degrees will be considered better mobility.

#### CRPS-specific severity

The CRPS severity score (CSS) will be used to comprehensively assess the cardinal features of CRPS-I (sensory, vasomotor, sudomotor, and motor/trophic changes) following the Budapest criteria, providing a disease-specific measure of severity (4).

#### Activities of daily living (ADL)

ADL will be assessed using the modified Barthel index (MBI), which covers domains such as feeding, dressing, toileting, continence, and mobility. Scores will range from 0 to 100, with higher scores indicating greater degrees of independence.

#### Thermal asymmetry

Thermal Asymmetry: Infrared thermography (FLIR E86) will be used to measure skin temperature at 3 regions (distal forearm, palm, and fingertips) of the affected and unaffected upper limbs. Thermal asymmetry is defined as ΔT ≥ 1.5 °C between homologous regions (39). To minimize confounding, measurements will be conducted in a temperature-controlled room (24 ± 1 °C) after a 20-min acclimatization period, during which the participants will rest in a seated position with both upper limbs exposed. Participants will be instructed to abstain from caffeine, smoking, and strenuous exercise for at least 2 h before assessment. Emotional state will be briefly recorded at the time of measurement.

#### Neuroplasticity correlates

Neuroplasticity correlates: Resting-state fMRI (3 T Siemens Prisma) will be analyzed using DPABI v6.0. Seed-based functional connectivity will focus on the anterior cingulate cortex (MNI coordinates: *x* = 0, *y* = 30, *z* = −10) and insula (*x* = 38, *y* = 20, *z* = −6), with connectivity strength calculated as Pearson’s correlation coefficients (29).

### Exploratory outcomes

#### Blinding

The success of blinding will be assessed using Bang’s blinding index (BI), which measures participants’ guesses of their group allocation at each time point.

#### Adverse events (AEs)

The safety profile will include AEs recorded following CTCAE v5.0. AE analysis will focus on incidence, severity, and causality (classified as definite, probable, or possible).

#### Rescue medication handling

The use of rescue medication (Celebrex) will be quantitatively recorded at each assessment session, incorporated as a covariate in linear mixed models to control confounding. It includes sensitivity analyses excluding high-frequency users (>3 doses/week).

#### Sample size

Sample size was calculated using G*Power 3.1.9.7 software (40), based on the primary outcome measure of pain intensity assessed by the VAS. Key parameters were set as follows: a minimum clinically important difference (MCID) of 1.5 points on the VAS, a standard deviation (SD) of 1.8 points derived from pilot data, a two-tailed significance level (*α*) of 0.05, and a statistical power (1 − *β*) of 90%. For the three-arm parallel design, which is meant to be analyzed using one-way analysis of variance (ANOVA), an effect size (f) of 0.42 (classified as Cohen’s medium-to-large effect) was estimated (25). The initial calculation indicated a requirement of 33 participants per group. To account for a conservative dropout rate of 20%, resulting in a final allocation of 42 participants per group (total *N* = 126).

#### Recruitment

Recruitment posters for this study will be prominently displayed at the outpatient clinic of Nanbu County People’s Hospital. In addition, the recruitment advertisement will be posted on the official WeChat public account of Nanbu County People’s Hospital to expand the audience and increase awareness. During the design phase of this trial, consultation will be conducted with representatives of patients with post-stroke pain to optimize the inclusion criteria.

#### Risk–benefit assessment

The potential benefits for participants and society include improved patient conditions and access to free medical care and condition assessments during the trial.

Risks to participants comprise treatment administration or withdrawal-related risks and the inherent risks of the disease. To protect participants’ health and interests during the trial, the researchers may discontinue the trial when necessary based on combination therapy/medication rules and discontinuation/withdrawal criteria, to protect participants’ health and interests.

#### Allocation of interventions

Participants will be randomized in a 1:1:1 ratio to the experimental group (YLDJJ acupuncture + rTMS), control group 1 (conventional acupuncture + rTMS), or control group 2 (sham acupuncture + rTMS). Block randomization with a fixed block size of 6 will be used via the blockrand package in R Statistical Software (v4.5.0). Each block will include 2 participants per group to maintain balanced assignments during recruitment. An independent statistician will manage the allocation sequence using sequentially numbered, opaque, sealed envelopes. Concealment will be maintained until intervention assignment to prevent disclosure to enrollment or assessment staff. After generating the randomization sequence, the independent clinical assessment center will directly notify the assigned intervention therapist of the randomization number and treatment group of each participant. Notifications will occur via encrypted text messages or secure WeChat, ensuring the integrity of allocation.

#### Blinding and unblinding procedures

The rTMS devices used in both the experimental and control groups will be identical in appearance and operational sounds. Needles of the same specification (0.30 mm × 40 mm) will be used in the manual acupuncture group. Only the acupoints will differ between groups, thereby preserving blinding integrity.

Participants, outcome assessors, and statisticians will remain blinded to group assignment. Blinding will be ensured by using uniform rTMS devices and procedures across all groups, and acupuncture sessions will be conducted in private rooms to prevent participants from observing treatments administered to other groups. Practitioners performing YLDJJ or conventional acupuncture will not be blinded due to the technical requirements of the protocols. To minimize potential bias, they will adhere to standardized communication scripts, avoid discussing treatment details with participants, and will not participate in outcome assessments.

If unblinding becomes necessary after the participant’s withdrawal, the treating investigator will request allocation disclosure from the Clinical Evaluation Center. The reason for withdrawal, the revealed allocation, and the involved personnel will be documented. Full formal unblinding will be done by an independent statistician after trial completion, final follow-up, database lock, and preliminary analysis. Sealed envelopes will be employed, and the unblinding process will be thoroughly documented.

#### Data collection and management

An electronic data capture (EDC) system will be developed for data collection and management. Pre-validated case report forms (CRFs) will be used to collect demographic information, clinical outcomes, AEs, and adherence to the protocol. Data will be collected longitudinally at baseline, on the 10th and 20th days of treatment, and at 1 and 3 months after treatment. The data entry process will begin with built-in checks and logical validations in the EDC system to reduce errors. Trained personnel will conduct double-entry validation. Custom scripts in the R language will be used to automatically conduct logical checks for outliers and inconsistencies. A three-stage coordination process will be adopted: outliers or missing data will be automatically detected, 10% manual comparisons between CRFs and the database will be randomly conducted, and senior data managers will arbitrate unresolved issues. All data will be stored on a password-protected server, using role-based access control. Patient identifiers will be anonymized through a unique study code. The main list will be stored separately, and daily encrypted cloud backups will be made. The anonymous dataset will be archived for ≥5 years after publication. Access to the original data needs approval from the principal investigator and the ethics committee. The database will be locked after blind review by an independent group. Before locking, all data queries will be resolved, the final analysis population will be determined, and the statistical analysis plan will be consistent with the protocol. After signing the data lock certificate, read-only access will be activated.

#### Quality control

Before its implementation, the trial protocol will be subjected to independent expert review, including experts in acupuncture rehabilitation, orthopedics, biostatistics, clinical methodology, etc., to ensure its scientific validity. All researchers will receive mandatory training covering patient screening, standardized acupuncture techniques, and EDC operations to ensure consistent operations. Independent auditors will conduct regular on-site supervision, verifying the protocol’s implementation, randomly selecting 10% of the clinical report forms for review, and promptly reviewing AE records. The EDC platform will be employed to ensure data quality through detailed modification audit records, automatic logical checks for outliers, and role-based access control to prevent unauthorized editing. Additionally, a three-level data monitoring committee will be established to ensure data integrity and participant safety. The primary monitor will be responsible for real-time program supervision during the intervention period; the secondary monitor (ethics and compliance experts) will review informed consent documents, track AES following CTCAE v5.0 standards, and ensure timely reporting of serious AEs to the TSC. The tertiary monitor (biostatisticians and data managers) will verify the dataset through double-entry validation and automated logical checks, resolve discrepancies, and ensure compliance with the pre-defined statistical analysis plan.

#### Confidentiality and ethics compliance

Patient privacy will be preserved by anonymizing all records. Data sharing will comply with ethical standards, and de-identified datasets will be accessible upon reasonable requests after publication.

#### Statistical methods

All statistical analyses will be conducted by an independent statistician blinded to group allocation. A two-sided significance level of *α* = 0.05 will be applied for all inferential tests.

#### Analysis populations

The intention-to-treat (ITT) population will include all randomized participants.

The per-protocol (PP) population will comprise participants who complete at least one intervention session and provide primary outcome data at the end of treatment (day 20).

#### Handling of missing data

Missing data will be addressed using multiple imputation by chained equations (MICE), producing 20 imputed datasets. Sensitivity analyses will be conducted to compare the results from the imputed datasets with those from complete-case analyses to assess robustness.

#### Descriptive statistics

Baseline characteristics will be summarized as follows:

Normally distributed continuous variables (e.g., age and baseline VAS score) will be expressed as mean ± standard deviation (M ± SD) and compared using one-way ANOVA to assess homogeneity across the three groups before intervention.

Non-normally distributed continuous variables will be reported as medians and compared using the Kruskal-Wallis test.

Categorical variables (e.g., sex and stroke type) will be presented as frequencies and compared using *χ*^2^ or Fisher’s exact test.

To control for its potential confounding effect, pre-stroke hand dominance will be recorded and included as a covariate (categorical variable: right-dominant, left-dominant) in the linear mixed-effects models for outcomes in which functional compensation is a key factor, such as the modified Barthel index (MBI), to control for its potential confounding effect.

#### Primary outcome analysis

Pain intensity, measured based on the VAS, will be analyzed using a linear mixed-effects model (LMM). Fixed effects will include group, time, group-by-time interaction, baseline VAS score, and rescue medication use. Random effects will account for participant-specific intercepts. Between-group differences at each time point will be estimated with 95% confidence intervals (CIs). A reduction of ≥1.5 points from baseline will be considered clinically meaningful. Pre-planned contrasts will be conducted to test the following hypotheses: (1) the experimental group (YLDJJ + rTMS) will be superior to control group 2 (sham acupuncture + rTMS); and (2) the experimental group will be superior to control group 1 (conventional acupuncture + rTMS). These comparisons will directly test the specific efficacy of YLDJJ acupuncture with those of both a sham control and a conventional acupuncture active control.

#### Secondary outcomes analysis

Continuous outcomes (CRPS severity score, FMA, MBI, infrared thermography ΔT, and rs-fMRI connectivity) will be analyzed using the same LMM specification as the primary outcome.

Ordinal outcomes (Brunnstrom stages and MAS) will be analyzed using generalized estimating equations (GEE) with a cumulative logit link function.

Categorical outcomes (e.g., AE adverse event causality) will be compared using *χ*^2^ or Fisher’s exact test.

#### Exploratory and safety analyses

Blinding success will be assessed using Bang’s BI with 95% CIs. The incidence of AEs will be compared using *χ*^2^ tests, and the severity of AEs will be analyzed via GEE.

Rescue medication use will be included as a covariate in all primary and secondary analyses. Sensitivity analyses will exclude high-frequency users (>3 doses per week).

#### Subgroup and sensitivity analyses

Subgroup analyses will be conducted based on stroke type (ischemic vs. hemorrhagic) and baseline NIHSS score (≤8 vs. >8). Sensitivity analyses will include comparisons between ITT and PP populations and the use of non-parametric alternatives if model assumptions are violated.

#### Software and reporting

All analyses will be conducted using R software (v4.5.0) and the packages lme4, mice, and geepack. All results will be reported as statistical estimates with 95% confidence intervals, along with effect sizes for statistically significant outcomes. The false discovery rate will be used for multiple comparison adjustment in secondary outcomes.

#### AE reporting and harms

AE documentation will follow CTCAE v5.0 for all events. Specific procedures for managing common AEs are as follows: For acupuncture, minor bleeding or hematoma will be managed with prolonged local pressure; syncope (needle fainting) will be managed by immediately withdrawing needles, placing the participant in a supine position, and monitoring vital signs. For rTMS, headaches will be managed with rest and non-opioid analgesics if necessary (recorded as rescue medication); in case of a seizure (despite excluding high-risk participants), stimulation will be stopped immediately, standard seizure first aid will be administered, and medical support will be sought. Expected AEs based on the literature and our pilot data include mild (19, 41), transient headache with rTMS (~10 to 15% incidence), and minor local bleeding/bruising with acupuncture (~5 to 10% incidence). For the primary safety analysis, the focus will be on clinically significant risks by including only grade ≥2 (moderate to severe) AEs. Serious adverse events (SAEs) will be events causing death, permanent/significant disability, permanent organ damage, or hospitalization/prolonged stay. In case of SAEs, researchers will immediately ensure participant safety and report it to the sponsor and ethics committee within 24 h. The trial center and blinded editor will jointly perform emergency unblinding due to SAEs. Researchers will determine the need for further treatment using unblinded information.

#### Plans for communicating important protocol amendments to relevant parties

Protocol modifications require approval from the study organizer, ethics committee, and clinical research center. Furthermore, such modifications must be updated in the clinical research registration center.

#### Dissemination plans

Trial results will be published in peer-reviewed journals and shared with the media and public.

## Discussion

Our trial specifically targets patients 3–12 months after stroke, a period encompassing the late subacute to early chronic recovery phases (42). This window corresponds to the established consolidation of CRPS-I, where initial post-stroke changes evolve into sustained central sensitization and maladaptive neuroplasticity (14, 43). This timeframe aligns with the peak incidence of the syndrome (4), allowing us to intervene when the underlying pain mechanisms fully manifest but are potentially still modifiable. We hypothesized that our dual-pathway intervention, combining central neuromodulation (rTMS) with systemic peripheral regulation (YLDJJ acupuncture), can ideally disrupt this entrenched pathophysiology at this critical juncture.

The YLDJJ technology allows sequential acupuncture at the Yuan (Source) and Luo (Connecting) points along all 12 meridians, specifically targeting peripheral and central sensitization pathways. This method may be more effective than traditional local acupoint acupuncture in enhancing the release of endogenous opioid substances, inhibiting the cascading reactions of neuroinflammation, and downregulating Reg3b expression in dorsal root ganglia (26, 28, 44). rTMS modulates cortical excitability, suppresses thalamic-cortical rhythmic disorders, and promotes adaptive plasticity (45, 46). When combined with acupuncture, this dual intervention breaks the triad of CRPS-I. This triad includes neuroinflammation, autonomic dysfunction, and maladaptive changes. This dual-pathway strategy is consistent with emerging evidence supporting multi-system interventions over single treatments for complex pain syndromes.

Compared to pharmacotherapy, this protocol offers a non-pharmacologic alternative with a lower risk of systemic side effects. The 20-day intensive regimen leverages rTMS-induced neuroplasticity (47), while acupuncture sustains effects through neurohumoral regulation and corticospinal tract remodeling (48). Blinding procedures such as standardized rTMS setup, and private acupuncture rooms, minimize performance bias. Inclusion of objective measures, such as rs-fMRI and infrared thermography, will elucidate neurophysiological correlates of response, advancing mechanistic understanding beyond symptom relief.

This trial has several limitations that warrant consideration. First, although the use of a sham acupuncture plus rTMS control group more effectively isolates the specific effects of acupuncture and maintains blinding, it cannot reflect the independent efficacy of rTMS. Second, while the 3-month follow-up period is sufficient for assessing short-to-medium-term efficacy, it may be insufficient for evaluating long-term durability and recurrence rates in patients with CRPS-I who suffer from chronic fluctuating disease progression. Furthermore, if the study confirms efficacy in the enrolled population, caution is warranted when extending its use to high-risk elderly cohorts (≥55 years old). In such cohorts, the incidence of stroke doubles every decade (2) and often involves several medications, frailty, and other comorbidities, factors that may significantly affect the response to treatment. Finally, this study excluded patients with severe aphasia or cognitive impairment, who represent a vulnerable subgroup prone to CRPS-I after stroke (4). This underscores the need for tailored protocols in clinical practice.

To fill these research gaps, future studies should focus on the following directions: considering age-stratified recruitment (e.g., ≥55 years vs. <55 years) to evaluate efficacy in high-incidence subgroups and optimize dose–response parameters for elderly patients; extending the follow-up period to ≥12 months, especially for the elderly group prone to late recurrence, to systematically assess the persistence of pain relief and functional recovery; developing adaptive protocols for excluded populations, such as simplified acupuncture or rTMS protocols for patients with severe aphasia; and using neuroplasticity biomarkers to personalize stimulation targets. In addition, cost–benefit analysis should be integrated to compare the economic efficiency of YLDJJ combined with rTMS and pharmacological treatment, especially in regions with limited resources and a continuously increasing burden of stroke in the elderly population (1); Finally, as predictors of efficacy, serum neuroinflammatory markers, such as IL-1β, and TNF-*α*, should be verified to provide a basis for phenotypic mechanism stratification of CRPS-I in the elderly. If effective, YLDJJ+rTMS may become a level B recommendation for post-stroke CRPS-I, addressing the unmet needs in resource-limited settings.
